# Our New Associate Editor

**Published:** 1916-09

**Authors:** 


**Our New Associate Editor.** In accordance with the policy of the **Buffalo Medical Journal,** to afford adequate representation of the professional interests of all western New York, Syracuse has been added to our list of centers of editorial supervision. We are most fortunate in securing the services of Dr. J. J. Buettner, a native of Syracuse, and educated in the schools and University of that city. After receiving the degree of M. D. from the Medical Dept. of Syracuse University in 1896, Dr. Buettner studied in Berlin, Vienna and London, and returned to Syracuse to engage in medical practice. He is a member of the Syracuse Academy of Medicine, the A. M. A. and its state and county affiliating societies; the New York, American and Interstate Societies of Anaesthetists; and has been for some years Secretary of the Central New York Medical Association. His practice has been, for some years, limited to anaesthesia and he is anaesthetist to the Syracuse Hospital for Women and Children, St. Joseph's Hospital and the Syracuse Free Dispensary.

